# A generalizable one health framework for the control of zoonotic diseases

**DOI:** 10.1038/s41598-022-12619-1

**Published:** 2022-05-21

**Authors:** Ria R. Ghai, Ryan M. Wallace, James C. Kile, Trevor R. Shoemaker, Antonio R. Vieira, Maria E. Negron, Sean V. Shadomy, Julie R. Sinclair, Grace W. Goryoka, Stephanie J. Salyer, Casey Barton Behravesh

**Affiliations:** 1 National Center for Emerging and Zoonotic Infectious Diseases, Centers for Disease Control and Prevention, Atlanta, USA; 2 National Center for Immunization and Respiratory Diseases, Centers for Disease Control and Prevention, Atlanta, USA; 3 Food and Agriculture Organization of the United Nations (FAO), Rome, Italy; 4 Center for Global Health, Centers for Disease Control and Prevention, Atlanta, USA

**Keywords:** Epidemiology, Infectious diseases

## Abstract

Effectively preventing and controlling zoonotic diseases requires a One Health approach that involves collaboration across sectors responsible for human health, animal health (both domestic and wildlife), and the environment, as well as other partners. Here we describe the Generalizable One Health Framework (GOHF), a five-step framework that provides structure for using a One Health approach in zoonotic disease programs being implemented at the local, sub-national, national, regional, or international level. Part of the framework is a toolkit that compiles existing resources and presents them following a stepwise schematic, allowing users to identify relevant resources as they are required. Coupled with recommendations for implementing a One Health approach for zoonotic disease prevention and control in technical domains including laboratory, surveillance, preparedness and response, this framework can mobilize One Health and thereby enhance and guide capacity building to combat zoonotic disease threats at the human–animal–environment interface.

## Introduction

One Health is a collaborative, multisectoral, and transdisciplinary approach—working at the local, national, regional and global levels—with the goal of achieving optimal health outcomes that recognize the interconnection between people, animals, plants, and their shared environment. In recent decades, the One Health approach has gained traction in combatting health issues at the human–animal–environment interface. Zoonotic diseases, infectious agents shared between animals and people, are a formidable challenge in One Health. Evolving conditions at the human–animal–environment interface due to factors like climate change, land use change (e.g., deforestation and agricultural intensification) and increasing travel and trade have directly and indirectly affected the emergence and reemergence of zoonotic diseases^1–3^. Applying a One Health approach to optimize zoonotic disease prevention and control programs can save lives by improving efficient use of resources (finances, infrastructure and personnel) and the quality and timeliness of healthcare delivery^4–8^. Despite increasing awareness of the One Health approach, lack of communication and coordination between human health, animal health, and environment sectors can still hinder implementation. Internationally, the Tripartite organizations, namely the Food and Agriculture Organization of the United Nations (FAO), the World Organisation for Animal Health (OIE), and World Health Organization (WHO), have exemplified using a multisectoral, One Health approach through mandated inter-agency collaboration^9^, and endorsement of One Health to facilitate sustained collaboration for zoonotic disease control at the local, subnational, national, regional, and international level through the guide, “Taking a Multisectoral, One Health Approach: A Tripartite Guide to Addressing Zoonotic Diseases in Countries” (hereafter the Tripartite Zoonoses Guide or TZG)^10^.

**Figure 1:**
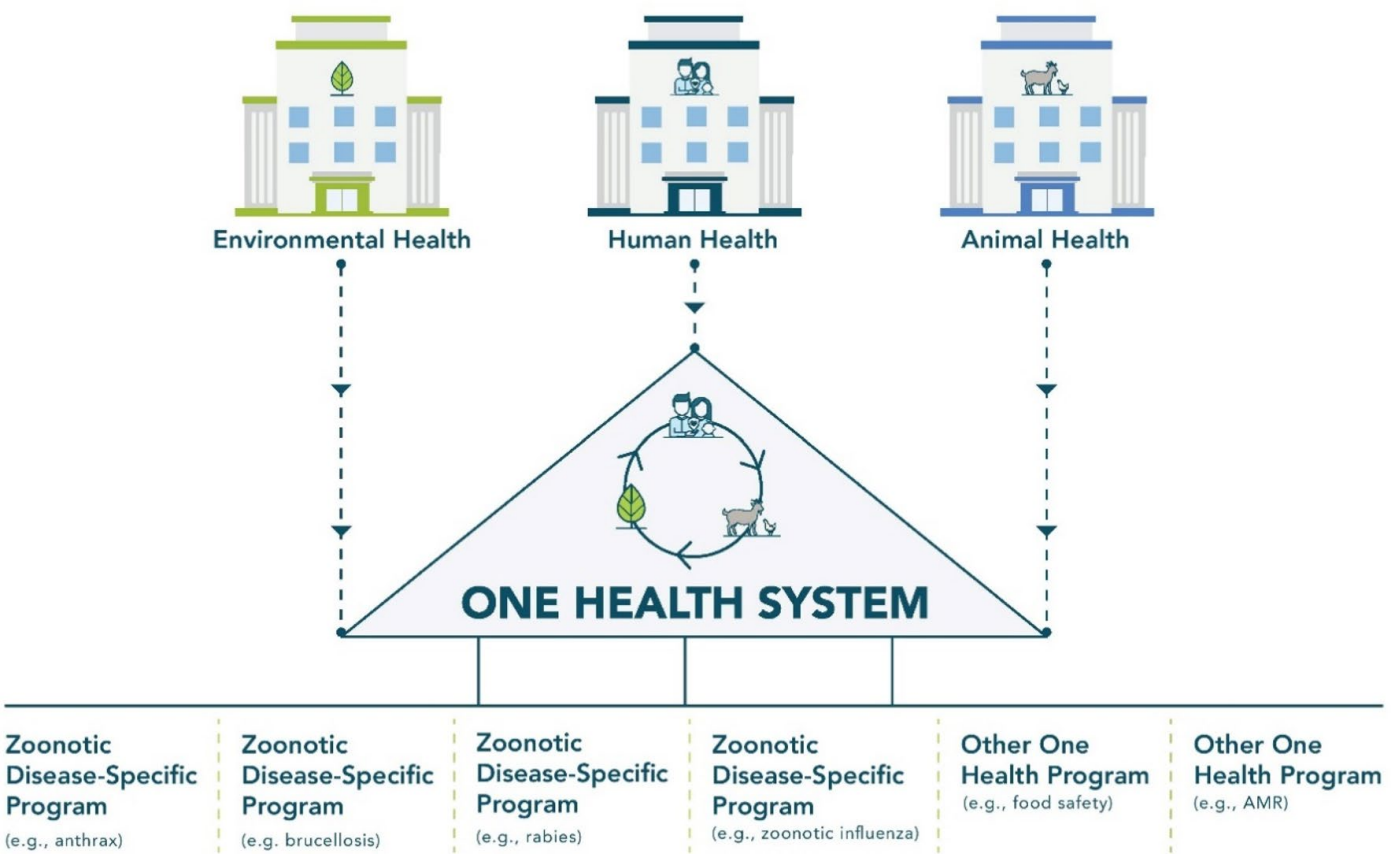
One Health systems versus zoonotic-disease specific programs. Zoonotic disease-specific programs are generally programs with a focus on a specific pathogen, disease complex, syndrome, or subject. Zoonotic-disease specific programs often include One Health activities, but tend to be led by a specific sector (e.g., human health, animal health). In contrast, a One Health system often includes delegates from all relevant One Health sectors (e.g., human, animal and environmental health) and coordinates all One Health activities, including zoonotic-disease specific programs, across participating sectors. *AMR* antimicrobial resistance.

A One Health approach can be applied broadly to support overarching systems that improve multisectoral, One Health coordination, or the approach can be applied to specific topics, such as antimicrobial resistance, climate change, zoonotic disease control, or food safety and security. A systems-based One Health approach (Fig. 1) often involves development of multisectoral, One Health coordination mechanisms (OH-MCMs)^10^. OH-MCMs can create a way to coordinate all One Health activities across all relevant sectors^10^. While One Health systems such as OH-MCMs are not necessarily specific to zoonotic diseases, they may directly oversee zoonotic disease programs, or indirectly bolster associated One Health coordination. The TZG primarily provides guidance for a systems-based One Health approach, while providing examples from specific programs. While One Health systems-based approaches are an effective method of building sustainable coordination and collaboration across sectors^10^, initiating coordination through a zoonotic disease-specific program (Fig. 1) that uses a One Health approach to initially focus on a few key priority diseases may be more tractable in the short term. Here, we utilize the approach established in the TZG, and apply these lessons to programs, specifically control of zoonotic pathogens.

**Figure 2:**
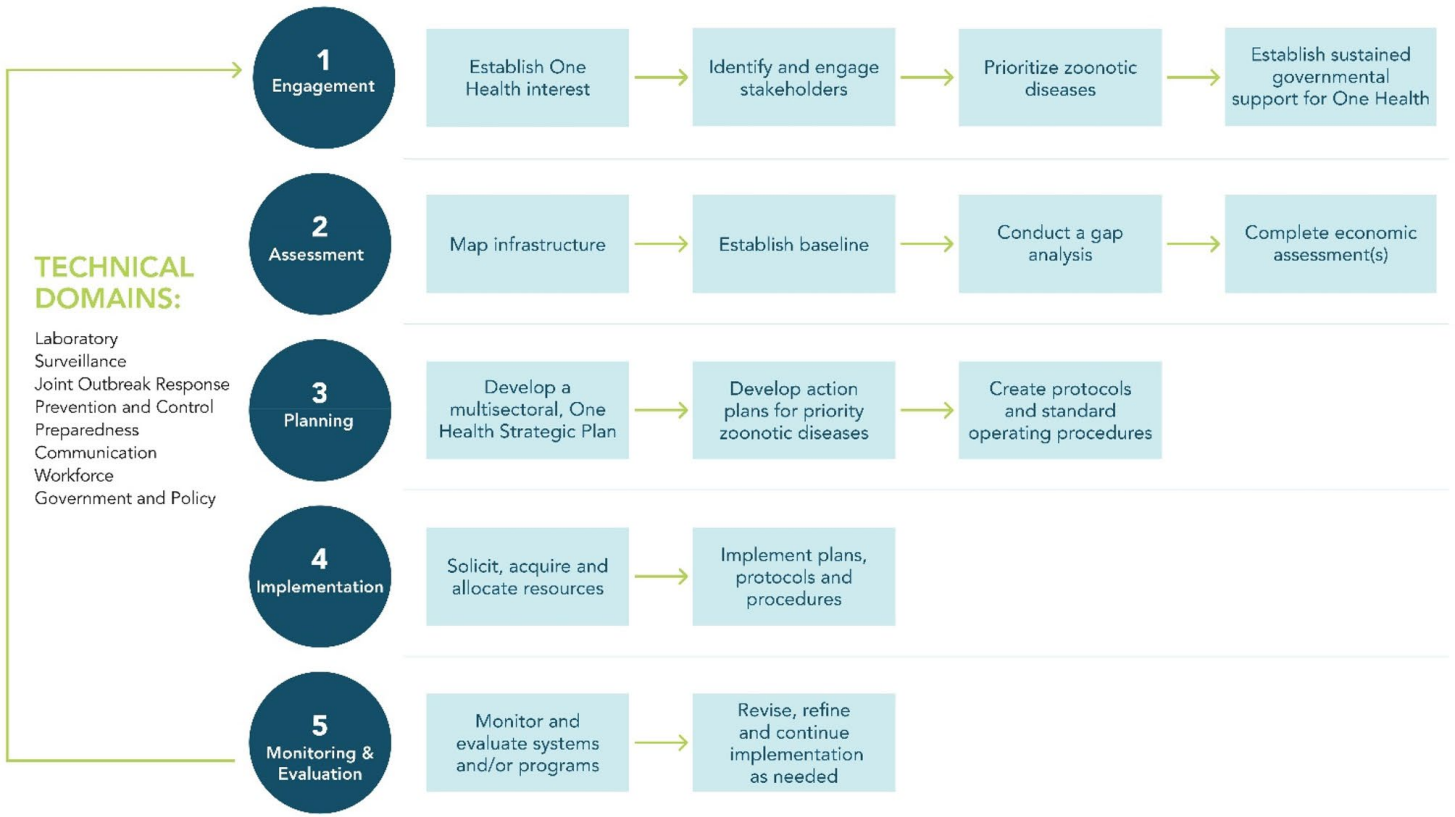
Generalized One Health framework visualization. Dark teal circles indicate stepwise headings. Light blue boxes indicate activities under these headings that pertain to building a One Health system or zoonotic disease-specific program. Technical domains pertain to all steps within the GOHF, and often comprise essential elements of a successful One Health system or zoonotic disease-specific program to be addressed.

The Generalized One Health Framework (GOHF) presented here provides recommendations for how to use a One Health approach to improve multisectoral collaboration and thereby enhance the prevention and control of zoonotic diseases. The GOHF includes a visualization (Fig. 2) that presents a series of five steps and corresponding activities which provide structure for how countries may develop capacity to coordinate zoonotic disease programming across sectors. The objectives and outcomes intended for each step are listed in Table 1. The GOHF was developed primarily for use by governmental officials in public health, animal health, or environment sectors working on One Health and zoonotic disease programming at the local, subnational, national, or international levels. Users of the GOHF may choose to enter this framework at any step based on their current capacity, although a numerical order is proposed for ease of use. The GOHF also includes a toolkit (see Supplementary Tables S1–5), which compiles available resources matched to each step and activity within the framework. To add context for application of the GOHF, we include zoonotic disease examples as they have been applied throughout the globe. The GOHF is not intended to be prescriptive—rather, it is meant to be broadly applicable to common zoonotic diseases in most settings, including use within both high-income and low-middle income countries, and ranging from the local to the international level.

**Table 1:** Objectives and outcomes for each stage of the generalized pathway for zoonotic diseases.

	Objective	Outcome(s)
Step 1: engagement	Establish initial One Health collaborations around zoonotic disease control	A prioritized list of zoonotic diseases of greatest national concern Next steps and plans to address priority zoonotic diseases using a One Health approach Government commitment to using a One Health approach
Step 2: assessment	Understand limitations and disparities in resources within the One Health system and/or relevant One Health sectors	Improved understanding of the current situation Identified areas of strengths and weakness in each sector relevant to the One Health system or zoonotic disease program
Step 3: planning	Develop plans and protocols that include and leverage all relevant One Health sectors	Plans, protocols and procedures that are ready for implementation
Step 4: implementation	Implementation of programs that use a One Health approach	Investment in sustained One Health systems or zoonotic disease programs Operationalization of One Health systems or zoonotic disease programs
Step 5: monitoring and evaluation	Identify successes and improve upon weaknesses of One Health systems and/or programs	Improved capacity to control zoonotic diseases and other threats at the human–animal–environment interface

Finally, since effective action should involve application of the GOHF to all aspects of a zoonotic disease-specific program, we describe how the GOHF can be applied across several technical domains, specifically laboratory, surveillance, joint outbreak response, prevention and control, preparedness, communication, and government and policy (Fig. 2).

## Methods

The GOHF was developed by subject matter experts in One Health, zoonotic diseases, public health, and animal health at U.S. Centers for Disease Control and Prevention (CDC), and FAO. This framework was developed by combining successful existing and idealized processes for implementing zoonotic disease programming globally, leveraging subject-matter experts in anthrax, brucellosis, rabies, Rift Valley fever, and zoonotic influenza to provide example. Resources included in the Supplementary Tables are not an exhaustive list of all resources available; expert opinion, accessibility of the resource, and frequency of use were used to identify the most relevant resources available.

### Step 1: Engagement

#### Establishing One Health interest by identifying and engaging stakeholders

Whether developing a One Health systems-based or zoonotic disease-specific program (Fig. 1), the process begins with recognizing that a multisectoral, One Health approach can optimize resources and improve human, animal and environmental health outcomes^4–8^. Given these and similar advantages, an initial stage in developing One Health systems for zoonotic disease-specific programs involves exploring context-specific benefits, required modifications to current operations, and the level of interest and commitment expressed by stakeholders (Supplementary Table S1, 1.1, 1.2).

#### Identifying and engaging stakeholders

Early in system or program development, stakeholders from all relevant One Health sectors (i.e., public health, agriculture/livestock health, wildlife health, environment, and others) as well as other relevant fields (e.g., social and political sciences) must be identified^9^. Identifying stakeholders that represent all interests and levels early in the process can help build trust and improve sustainability (Supplementary Table S1, 1.3–1.5). In Kenya, for example, social network analysis not only identified relevant stakeholders involved in Rift Valley fever programs at the subnational and national level, but also identified the strength of collaboration and influence of each^11^, which helped determine the physical location to situate the OH-MCM^11^. Once appropriate stakeholders are identified, establishing roles and responsibilities (perhaps through formalized agreements such as Memorandums of Understanding or Letters of Agreement) between participating stakeholders can assist in establishing accountability and facilitating steady progress. Finally, to ensure that relevant stakeholders remain engaged as programs are expanded, combined, or re-organized, the process of identifying and including appropriate stakeholders should be routinely revisited.

#### Prioritizing zoonotic diseases

Often, resources are not adequate to address all needs for zoonotic disease control, which necessitates prioritizing zoonotic diseases for resource allocation. An objective, formalized prioritization process with equal participation and input from all relevant sectors will have the added advantage of helping to establish One Health commitment and collaborations^10,12,13^. To address the prioritization needs of countries, regions and other localities, the CDC developed the One Health Zoonotic Disease Prioritization process (OHZDP; Supplementary Table S1, 1.6). The OHZDP uses a transparent approach to prioritize zoonotic diseases of greatest concern for joint collaboration^12,13^. Collaborative prioritization promotes program ownership and uptake, and resource and information sharing between all participating stakeholders. Outcomes of the OHZDP and other prioritization efforts are associated with improved scores on international evaluations such as the Joint External Evaluation (JEE; Supplementary Table S2, 2.2), development of National Action Plans for Health Security (NAPHS; Supplementary Table S4, 4.1) and other One Health strategic plans, and enhanced zoonotic disease program capacity^14–17^.

#### Establishing sustained government support for One Health

Often, government support for zoonotic diseases may surge during outbreaks and wane in the absence of emergencies and crises-driven funding. However, several examples indicate that sustainable support entrenched in a government-led strategy to prevent and control zoonotic diseases is the cornerstone of a successful program^18–22^. In Thailand, an institutionalized One Health strategy was precipitated by the devastating public health and socio-economic effects of avian (H_5_N_1_) and pandemic (H_1_N_1_) influenza in 2004 and 2009, respectively. Growing partnerships around influenza created opportunities and demonstrated successes that promoted further collaboration, ultimately leading to a cabinet-endorsed resolution where One Health was a core principle, and formation of a Thai Coordinating Unit for One Health^21,22^. Published articles from Kenya^18,19^ and Egypt^20^ also chronicle the path to institutionalizing One Health within government and provide examples for establishing sustainable support. At minimum, government support for One Health systems and programs should include dedicated domestic resources (e.g., financial, infrastructure and personnel) and political will to initiate and sustain action^10,23^. For those advocating for increased governmental investment in One Health, one way to establish support is by demonstrating clear benefits of proposed activities at a reasonable cost, which can be accomplished through cost effectiveness analysis (Supplementary Table S1, 1.10). More formalized measures, such as advocacy or awareness campaigns, may be useful for communicating this information to higher levels of government. Such campaigns can be conducted for little or no cost using social media platforms.

### Step 2: Assessment

#### Mapping infrastructure

In order to develop realistic and achievable plans for One Health systems or zoonotic disease-specific programs, the available infrastructure must be understood^10^. Infrastructure mapping can help visualize mechanisms of informal and formal communication, and collaboration and coordination occurring within and between sectors in the form of a network map. By visualizing the network, infrastructure mapping can identify redundancy, gaps and weaknesses in the system or program being assessed^4,10^ (Supplementary Table S2, 2.1).

#### Establishing a baseline

In order to develop prevention and control plans that effectively channel resources, baseline information on the status of current activities, such as the burden of the zoonotic disease and its epidemiologic situation, should be established^24^. Analyzing disease-specific data from baseline studies and existing surveillance and laboratory activities at the local, sub-national, national, and regional levels may be a first step where such data exist. In countries where adequate data are not available, primary literature and unpublished findings from academic institutions or non-governmental organizations may also provide useful information. In some instances, new investigations such as serological surveys or pilot studies may be necessary to establish the baseline epidemiologic situation, such as the primary hosts and reservoirs, circulating species or strains, and prevalence in human and animal populations.

#### Conducting gap analysis

Once baseline information is understood, it is possible to identify gaps in current capacity within and between sectors responsible for managing the system or program. Unlike infrastructure mapping which visually illustrates multisectoral coordination, gap analysis critically assesses technical capacity to achieve a goal. In some instances, tools for gap analysis provide stepwise guidance through the assignment of a score, allowing users to establish current and desired conditions (Supplementary Table S2, 2.2 and 2.3). Some zoonotic diseases also have tools that are specific to the pathogen, such as the Stepwise Approach towards Rabies Elimination (SARE; Supplementary Table S3, 3.6). In many ways, the SARE tool and paired Blueprint for Rabies Control (Supplementary Table 5, 5.6) exemplify zoonotic disease-specific guidance that embodies a One Health approach and have therefore been widely adopted in both country-level and regional plans to eliminate human deaths from dog-mediated rabies^25–28^.

#### Completing economic assessments

Perhaps the most compelling argument for investment in zoonotic disease prevention and control is the cost-effectiveness of the proposed program. Specifically, understanding how the economic burden of “status quo” (i.e., the cost of illness) compares to various scenarios of investment in control and elimination can both only improve the probability of program success and facilitate program endorsement by stakeholders and government. For zoonotic diseases, economic evaluations or decision analyses that account for all stakeholders are necessary to establish the societal cost of the disease as well as the benefits of prevention and control^29–31^. Indeed, while the cost savings of zoonotic disease prevention and control may not be readily apparent to a single sector, previous research has shown economic benefits to both government (in terms of cost savings) and society (in terms of reduced morbidity and mortality in humans and animals) in the case of brucellosis^32^, rabies^33^, and salmonellosis^31,34^ (Supplementary Tables S2, 2.11 and S3, 3.9).

### Step 3: Planning

#### Developing a multisectoral, one health strategic plan

If gaps or weaknesses are identified during the assessment phase, a formalized strategy to enhance collaboration across government can be articulated through strategic planning^10^. Strategic plans are often long-term (5–10 year), forward-looking documents that should be drafted and endorsed with equal input by all relevant sectors, and include a shared vision with achievable goals and objectives^35^. Several strategic planning resources exist to assist countries with developing One Health strategic plans, although there are also many countries that develop successful plans independently^19,36–38^. Some zoonotic diseases have specific strategic planning resources, such as the Rabies Practical Workplan, a component of the SARE which translates pending activities into actionable work plans (Supplementary Table S5, 5.5)^39^. As applicable based on membership, governments should also consider how their One Health strategic plans and specific zoonotic disease action plans (described below) may be integrated into international initiatives, such as the NAPHS (Supplementary Table S4, 4.1)^40^.

#### Developing action plans for priority zoonotic diseases

While action plans can be developed independently of a strategic plan, they can benefit from linkage. The goals and objectives developed during strategic planning can be used to develop activities in an action plan, thereby making goals and objectives implementable. Action plans can therefore serve as a roadmap for implementing the agreed upon vision of a collaborative One Health effort. Action plans typically highlight the short-term (1 year or less) activities that are required to achieve a mission. They outline the roles and responsibilities of all partners, and identify the resources needed to implement outlined activities^10^. Outcomes from prioritization exercises and disease-specific gap analysis exercises may also be used to inform the development of these action plans.

### Step 4: Implementation

#### Soliciting, acquiring and allocating resources

Officials that are preparing to implement developed plans should have an in-depth understanding of the tasks associated with building their One Health system or zoonotic disease-specific program. At this stage, costing the program identifies how available resources (including human, financial and physical) will be allocated. This information can assist with devising a strategy to obtain missing resources, such as launching advocacy plans, soliciting resources from private industry, or seeking non-traditional partners including the military, universities, or ministries not directly associated with health (e.g. education, finance, or tourism). Exploring whether programming fits under the mandates of international agencies or forming regional partnerships to garner international assistance may also be worthwhile depending on current or future global priorities. Ideally, sufficient resources to carry programs through to completion should be identified prior to implementation of each phase, as this can help avoid premature program terminations.

#### Implementing plans, protocols and procedures

Budgeted and financed plans allow One Health systems or zoonotic disease-specific programs to begin implementation. While this phase largely involves the progressive roll-out of programs resources exist to smooth or improve program implementation. Technological innovations including software and web platforms, mobile phones, tablets, and applications or “apps” are powerful resources being used to implement surveillance, prevention, control and preparedness activities. For example, in the United States, text-based monitoring has also been used to improve detection of illnesses caused by novel influenza A viruses^41^. More sophisticated smartphone technology has spurred comprehensive surveillance, data collection, and prevention applications. For example, the WVS Data Collection app (Supplementary Table S7, 7.1) uses a One Health approach through its Integrated Bite Case Management system for rabies to collect data on mass dog vaccination campaigns, community surveys, and hospital bite case management^42^. Similarly, the Kenya Animal Biosurveillance System (KABS) smartphone app expedites detection of wildlife and livestock zoonotic diseases. In 2017, the KABS reported cattle mortalities that ultimately identified anthrax that triggered a One Health investigation that assessed people, animals and contaminated environments^43,44^. Smartphone technology is now also being used for laboratory diagnostics. For example, a smartphone-based system for detecting H5N1 avian influenza in clinical patient samples has a two-folder higher detectability than traditional fluorescent strip readers, making it a sensitive and portable system for field-based diagnostics^45^.

Whether implementation mechanisms are conventional or innovative, the ultimate goal at this stage is to implement an effective system that minimizes resources while reaching intended outcomes, including reductions in human and animal morbidity and mortality^46^.

### Step 5: Monitoring and evaluation

#### Monitor and evaluate systems and/or programs

Although monitoring and evaluation is the last step in the GOHF, monitoring and evaluation should ideally be established during the planning phase in order to track implementation outputs and systematically evaluate the successes, challenges, scope, and scale of programs. With respect to One Health systems, evidence suggests that despite a rise in One Health systems in recent years, few report the use of standardized or systematic monitoring and evaluation frameworks to demonstrate the effectiveness of the One Health approach^47,48^. While evaluating the multi-faceted nature of a One Health system or zoonotic disease-specific program can be complex, a framework for monitoring and evaluation of One Health systems can provide evidence to support decision-making. Recent efforts have used common frameworks for monitoring and evaluation of One Health systems, but additional work is still needed to develop a standardized approach to monitor and evaluate One Health systems (but see Supplementary Table S8 for examples).

### Applying a One Health approach to specific zoonotic disease technical domains

Effective implementation of a One Health approach should involve integration into many, if not all, facets of a zoonotic disease program. In the below sections, we highlight how a One Health approach can be applied to several technical domains that are commonly a part of zoonotic disease programs: laboratory, surveillance and joint outbreak investigation, prevention and control, preparedness, communication, workforce, and government and policy.

#### One Health in laboratory systems

**Figure 3:**
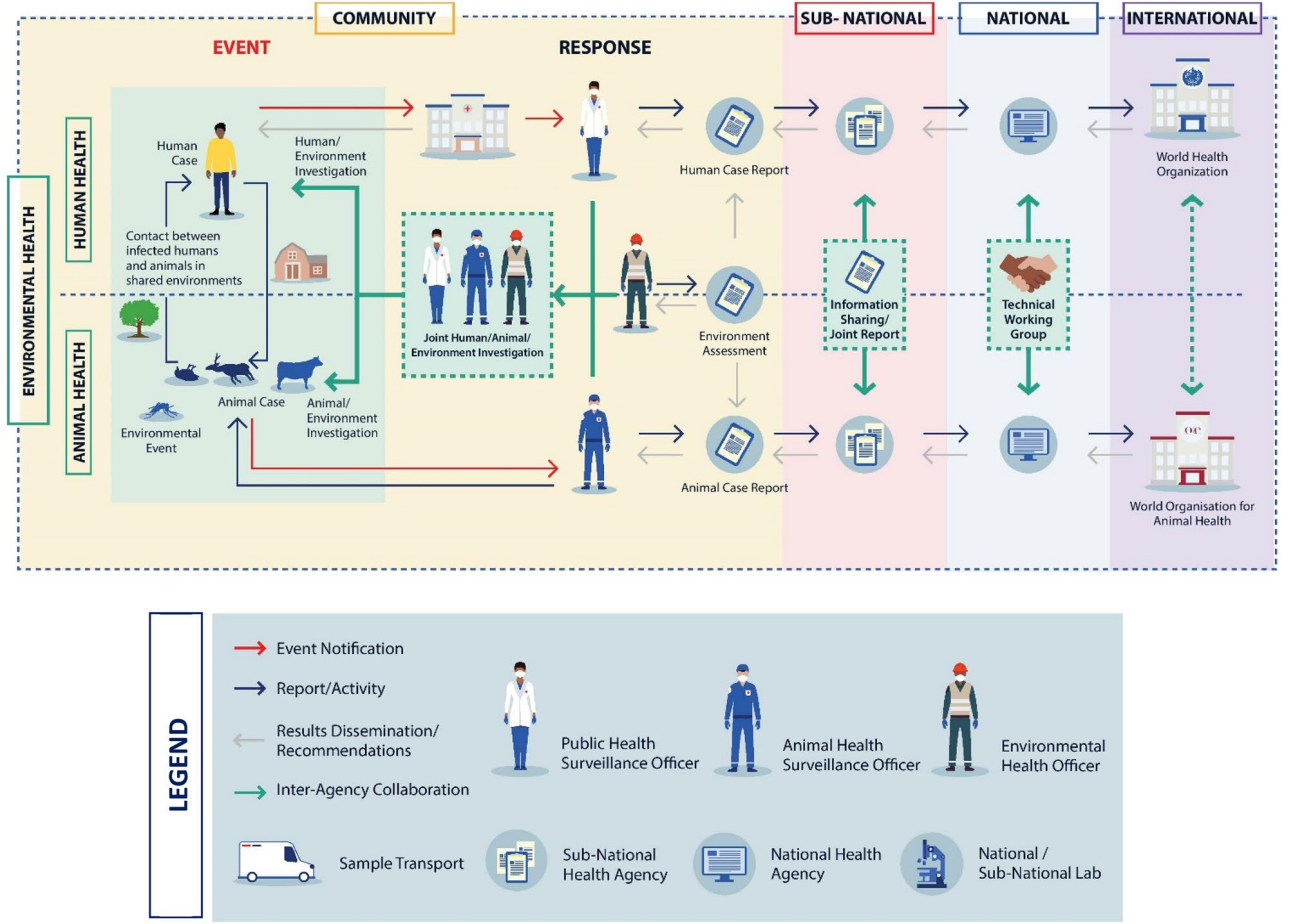
Taking a One Health approach to event-based surveillance. While each zoonotic disease requires a tailored surveillance strategy to ensure a targeted and expedient response, the One Health linkages (shown in teal) may be similar across many different event types. In this generalized zoonotic disease example, event-based surveillance begins with the detection of an “event” or verified signal in red that triggers a response. This event, detected at the community level, is reported to the sub-national (e.g., states, provinces, or jurisdictions), national and international levels from left to right. Results and recommendations are disseminated in the opposite direction (i.e., right to left), with the intention of communicating synthesized results and recommendations back to at-risk communities. See Supplementary Figs. S1–S5 for pathogen-specific examples of event-based surveillance in anthrax, brucellosis, rabies, Rift Valley fever, and zoonotic influenza viruses.

Central to any effective zoonotic disease prevention and control program is the ability to provide timely, accurate and reliable diagnostic testing to detect and characterize the pathogen within laboratory networks^24^. In many cases, however, public health, veterinary, and environment laboratories may work on the same One Health challenge without aligning methods, technologies, or analytical approaches; this can lead to duplication of effort. Implementing a multisectoral, One Health approach to laboratory systems can reduce program expenditures and improve response times through sharing of physical resources and/or data. When procedures to detect and diagnose a zoonotic pathogen are similar among human, animal and/or environmental samples, sharing resources or personnel may be beneficial. In Mongolia, for example, a program of exchanging information, experiences, and resources between veterinary and public health laboratories enabled veterinary laboratories to provide support during outbreaks of human anthrax and rabies that resulted in dramatic improvements in national diagnostic capacity^49^. Similarly in Canada, the Canadian Science Center for Human and Animal Health is the world’s first facility to have both human and animal Containment Level 4 labs together, allowing for cross-cutting laboratory research on zoonotic pathogens that have included Zika and Ebola^50^. In such instances, sector-specific laboratories may operate independently to detect and diagnose zoonotic pathogens, but laboratory protocols are aligned and standardized data are shared both within and across sectors to speed outbreak detection and identify sources of infection^51^ (Fig. 3 and Supplementary Figs. S1–S5). An example of success through laboratory data sharing is PulseNet, a US-based domestic and international network of food, animal, and public health laboratories where identifying enteric disease clusters of increasing incidence is estimated to have averted 270,000 foodborne illnesses and saved US $507 million each year^52–54^.

#### One Health in surveillance and joint outbreak investigation

Implementing a multisectoral, One Health approach to surveillance involves the systematic collection, coordination, and communication of data and reports between relevant sectors with the intent of providing accurate and complete information to inform decision-making^55,56^. In Fig. 3, we use event-based surveillance, which is the detection and reporting of “signals”, defined as information that may represent events of health importance, to highlight how a One Health approach can be used across a more generalized surveillance system to show coordination across sectors. At the community level, zoonotic disease events at the human-animal-environment interface may trigger joint or coordinated outbreak investigations that involve relevant human, animal and environmental health officials (Fig. 3 and Supplementary Figs. S1–S5)^57^. Jointly responding to outbreaks may reduce costs and foster collaboration between sectors^56^, as has been seen during investigations of monkeypox^58^, leptospirosis^59^, Rift Valley fever^60^, and anthrax and rabies^61^ (Fig. 3 and Supplementary Figs. S1–S5). Establishing coordinated investigation and response protocols across all participating sectors is a critical component of effective response plans^62^.

When surveillance data are compiled and analyzed by each sector separately, coordinated surveillance (through interoperable platforms or information sharing mechanisms) can facilitate data sharing between relevant One Health sectors. Collecting common, standardized data elements ensures that data from different sectors can be linked and analyzed together (Fig. 3 and Supplementary Figs. S1–S5). Coordinated surveillance can be used to identify early warning signals for emerging zoonotic disease, understand and monitor trends in the disease burden, and develop coordinated response activities^62^. A number of successful coordinated surveillance systems exist throughout the globe^56,63,64^, with the common theme being collaboration at the policy level, institutional level and operational level, as well as data and outcomes that are shared to the benefit of all participating sectors^65^.

#### One Health in prevention and control

While it seems evident that prevention and control programs that reduce the burden of disease in animal populations would correspondingly reduce the risk of human infection and disease (and vice versa), published records are limited to a few salient examples. For rabies, previous research has established that vaccination coverage of 70% or higher in dog populations can reduce the frequency of human dog-bite injuries, usage of post-exposure prophylaxis, and human rabies cases^66–70^. Further, evidence in epidemic-prone zoonotic diseases like influenza A viruses showed that animal vaccination prevented human outbreaks and perhaps also pandemics in China, where administration of a bivalent poultry vaccine eliminated human cases of H7N9^71,72^. Finally, for zoonotic pathogens with environmental stages, programs that reduce both animal and human exposure to contaminated environments can be more effective than single-sector disease measures^73^. While prevention and control plans may be specific to the zoonotic disease and the epidemiologic situation, these examples illustrate that taking a multisectoral, One Health approach to prevention and control can reduce the burden of disease while optimizing program resources.

#### One Health preparedness

A great deal of activity within the realm of emergency preparedness has focused on pandemic preparedness for emerging infectious diseases. Preparedness planning using a One Health approach involves participation, engagement and readiness from all relevant sectors through all stages of preparedness planning (Supplementary Tables S1–5). Improved coordination during emergencies may reduce the size or impact of a human pandemic, as was seen in the United States where the One Health linkages created during H5N1 planning and simulation are credited for the successful response to the swine-origin influenza H1N1 pandemic^74,75^. More generally, preparedness efforts that strengthen One Health coordination in laboratory, surveillance, and workforce (described in other sections) can benefit both routine and emergency activities.

#### One Health communication

An effective communication strategy should include activities both internal and external to the government. Internally within government, a communication strategy should establish process for relevant sectors and stakeholders to communicate and share information. Communication strategies can formalize channels and methods of communication, which helps align expectations, goals and messaging, as well as build relationships among internal One Health sectors. Despite the benefits of joint communication, cross-sector communication can be challenging for several reasons, including differences in terminologies used. Some tools, such as the One Health European Joint Programme Glossary, are available to identify and overcome terminology differences^76^. Alternatively, establishing joint communication at the start of an event can ensure synchronization from the outset. For example, in the United States, the One Health Federal Interagency COVID-19 Coordination Group was established at the beginning of the COVID-19 pandemic to share information across over 20 different governmental agencies and is the primary reason for harmonized messaging on the zoonotic nature of SARS-CoV-2 across the US government. Outside of government, a communication strategy can ensure that One Health stakeholders and partners receive united, consistent messaging that is unaffected by agency or mandate. Additionally, a communication strategy should guide public awareness campaigns, education on One Health issues of importance, and risk communication to best maximize public support and promote the uptake and success of any health program^62^.

#### One Health workforce

A number of international training frameworks highlight the need for training and education programs that use a One Health approach to equip the labor force with the skills necessary to combat zoonotic diseases (Supplementary Table S2, 2.2). Examples from several global programs with a focus on training health professionals using a One Health approach exist, including Field Epidemiology Training Programs (FETPs)^77,78^, the Global Laboratory Leadership Program (GLLP)^79^, the In-Service Applied Veterinary Epidemiology Training Programme (ISAVET)^80^, and the One Health Workforce project^81^. Through hands-on training, the cadre of health practitioners created through these and other One Health programs can amplify One Health-based training and curriculum messaging, are more likely to meet previously defined One Health Core Competencies^82^, and demonstrate improved ability to collaboratively respond to threats at the human–animal–environment interface^37,83^. Still needed are programs which include environmental health practitioners, incorporate curriculum on the role of climate and the environment on One Health issues, and formalize processes on when and how to engage environment sectors.

#### One Health in government and policy

Ensuring that the benefits of a One Health approach to zoonotic disease management are recognized by policy and decision makers can improve program success and sustainability. Institutionalizing One Health in governance is one means of establishing sustained support for programs that implement a One Health approach^74^. To this end, both the JEE and PVS Pathway (see Supplementary Table S2, 2.2 and 2.3) have legislative sections that are geared to assist in modernizing legislation to include a One Health approach. Typically, recognizing the need to institutionalize One Health in governance and policy occurs when a lack of coordination becomes apparent while addressing zoonotic disease threats, or when a gap in coordination capacity is identified during reporting or assessments^62^. Kenya is one such country that has published on its road to One Health institutionalization, and therefore provides an example of how governments may shift from sector-specific units or task forces to overarching governance through One Health systems^11,18,19,83,84^.

## Conclusions

This manuscript provides a framework for building capacity around zoonotic diseases using a One Health approach in a range of settings (Fig. 2). Users of the GOHF may identify their progress in developing coordinated zoonotic disease programs using the visualization and corresponding text of this manuscript, and then access resources to advance progress using the toolkit. The GOHF highlights that while developing prevention and control programs will require specialized technical expertise, the One Health approach taken is similar irrespective of the zoonotic disease. Further, rather than build independent programs for priority zoonotic diseases, this guidance is intended to deepen One Health capacity throughout the system. Therefore, ideally, a transition may occur from implementing a One Health approach for a few priority zoonotic diseases to gradually building a comprehensive One Health system that can combat a diversity of health threats, both endemic and emerging, at the human-animal-environment interface.

## Supplementary Information

Supplementary Figures.

Supplementary Tables.
