# Supplementary material for: A generalizable one health framework for the control of zoonotic diseases
Source: Sci Rep. 2022 May 21;12:8588. doi: 10.1038/s41598-022-12619-1 (PMC9124177; doi:10.1038/s41598-022-12619-1)
Supplement: Supplementary file 1 — Supplementary Figures. [file 41598_2022_12619_MOESM1_ESM.docx]

**Supplementary Figures – One Health Event-Based Surveillance Examples for Five Commonly Prioritized Zoonoses**

The figures below illustrate an event-based surveillance pathway for five zoonotic pathogens. Event-based surveillance was selected to represent the common One Health linkages (such as joint detection and investigation) across multiple pathogens because it provides a similar surveillance modality for comparison. It is recommended that a similar One Health approach to both event and indicator-based surveillance is considered where relevant for zoonotic disease surveillance systems (e.g. cross-sectoral data sharing and dissemination). Anthrax, brucellosis, rabies, Rift Valley fever, and zoonotic influenza viruses are depicted in each of five figures. These zoonoses were selected because they are commonly prioritized in One Health Zoonotic Disease Prioritization Workshops.

Each figure was developed in collaboration with CDC subject matter experts for each pathogen. Surveillance in each sector draws on the experience of subject matter experts, while One Health linkages (in teal) are idealized depictions of multisectoral coordination mechanisms that countries may endeavor to create. Each figure illustrates animal health (bottom; includes wildlife, livestock and companion animals) and human health (top) activities.

Event-based surveillance begins with the detection of an “event” or verified signal in red that triggers a response. This event, detected at the community level, is reported to the sub-national (e.g., states, provinces, or jurisdictions), national and international levels from left to right. Results and recommendations are disseminated in the opposite direction (i.e., right to left), with the intention of communicating synthesized results and recommendations back to at-risk communities.

Each zoonoses depicted differs in transmission mode, reservoirs, competent hosts, and symptomatic presentation in both people and animals. While each requires a tailored surveillance strategy to ensure a targeted and expedient response, the One Health linkages occurring in each case are similar. Some pathogens, such as anthrax and zoonotic influenza, can persist in the environment or contaminate habitats. Therefore, communication, collaboration and coordination with the environmental health sector should be sought when relevant. At the community level, communication and investigation across officers from the human, animal, and environmental health sectors can reduce the likelihood that events in animal populations do not result in human cases, and vice versa. At the sub-national and national levels, communicating compiled and analyzed data through meetings, briefs, or joint reports can improve incidence tracking, identify high-risk areas and activities for human-animal transmission, and facilitate continued multisectoral collaboration on disease control programming.

**Vignette Legend**


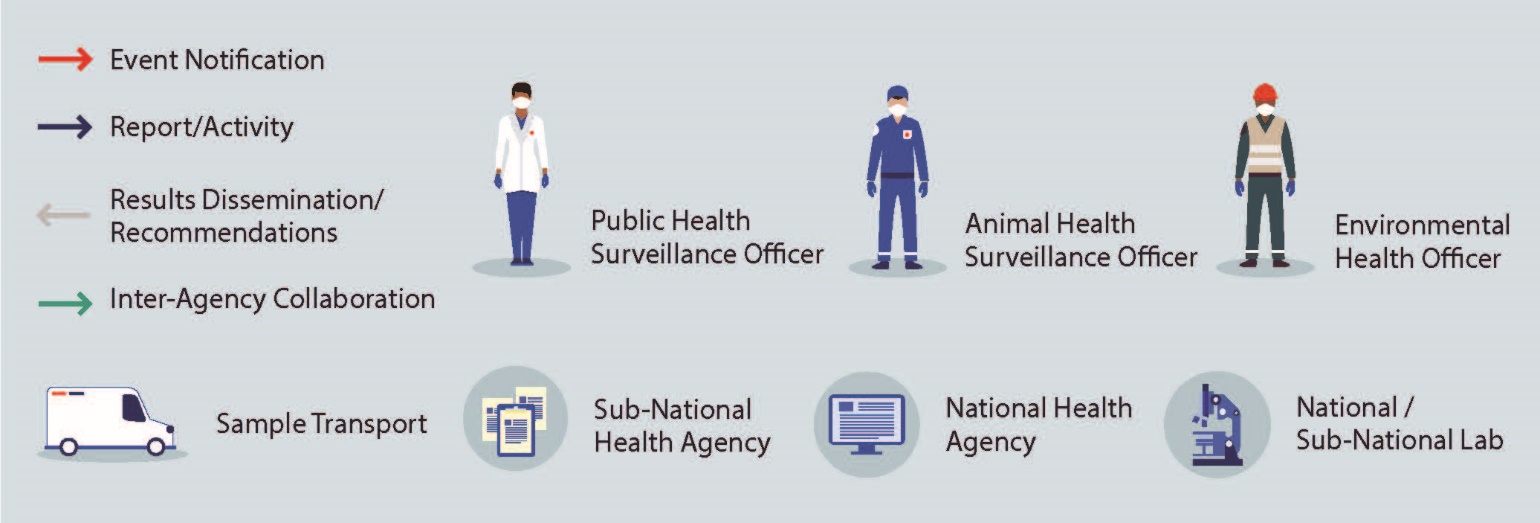


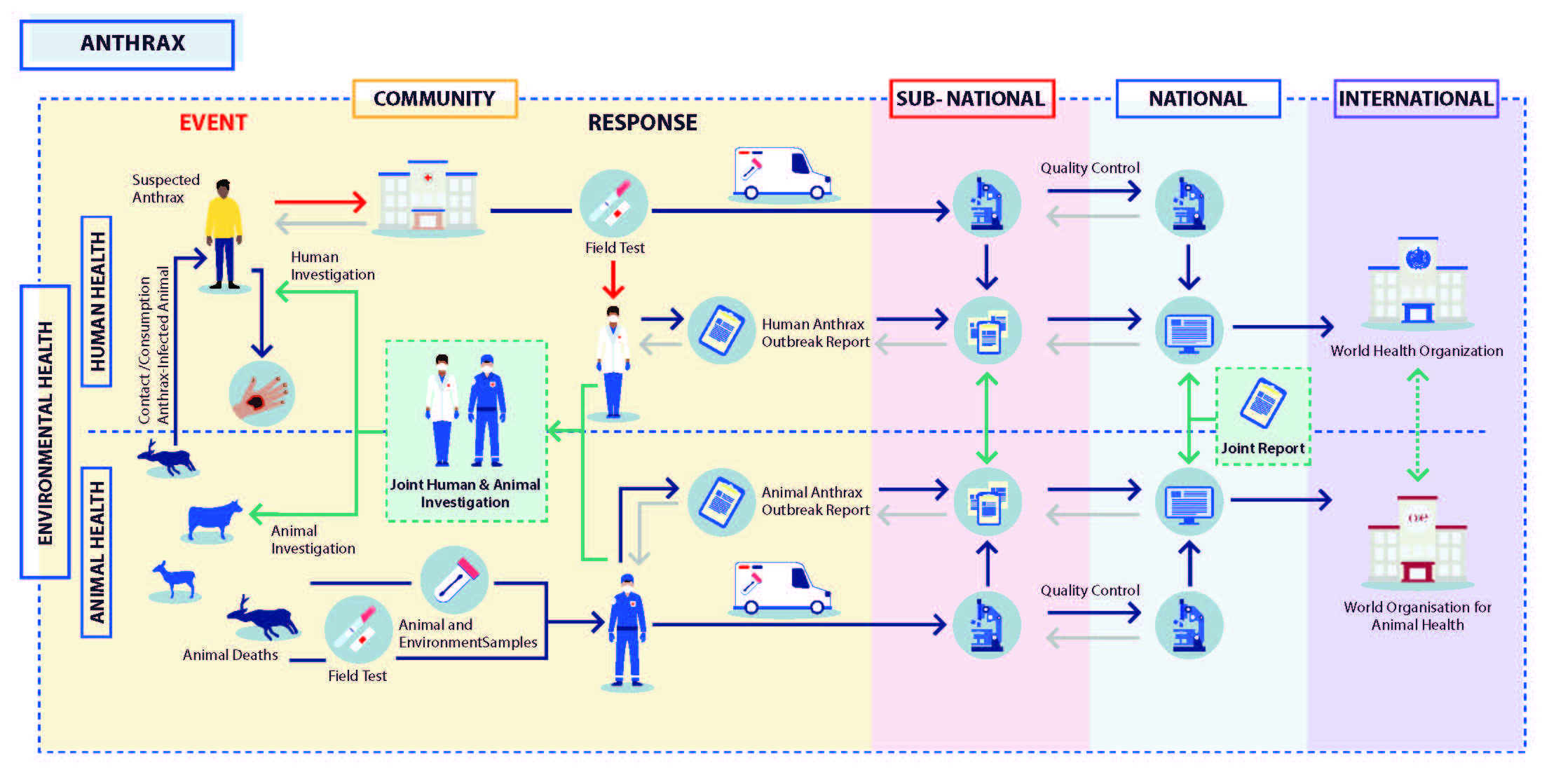


**Figure S1**. **Anthrax.** Anthrax is a zoonotic bacterial disease caused by *Bacillus anthracis*, which primarily infects herbivorous wildlife and livestock and is usually fatal in these animals. Human infections can result in a high mortality rate if not diagnosed and treated promptly. Anthrax outbreaks in humans are usually a consequence of interacting with anthrax-positive animals or animal products. Vaccinating livestock is the primary tool to prevent livestock infection and subsequent human infections. To control the health and economic impacts of anthrax, human, animal, and environmental health sectors can use a One Health approach to address ongoing outbreaks. It may be easier to obtain animal specimens for preliminary diagnostic testing for anthrax, and multisectoral sharing of the results of preliminary testing will enhance outbreak response. Finally, it is recommended that surveillance data is shared between sectors at the sub-national and national levels to ensure that anthrax surveillance, prevention and control programs are developed with data from all relevant sectors.


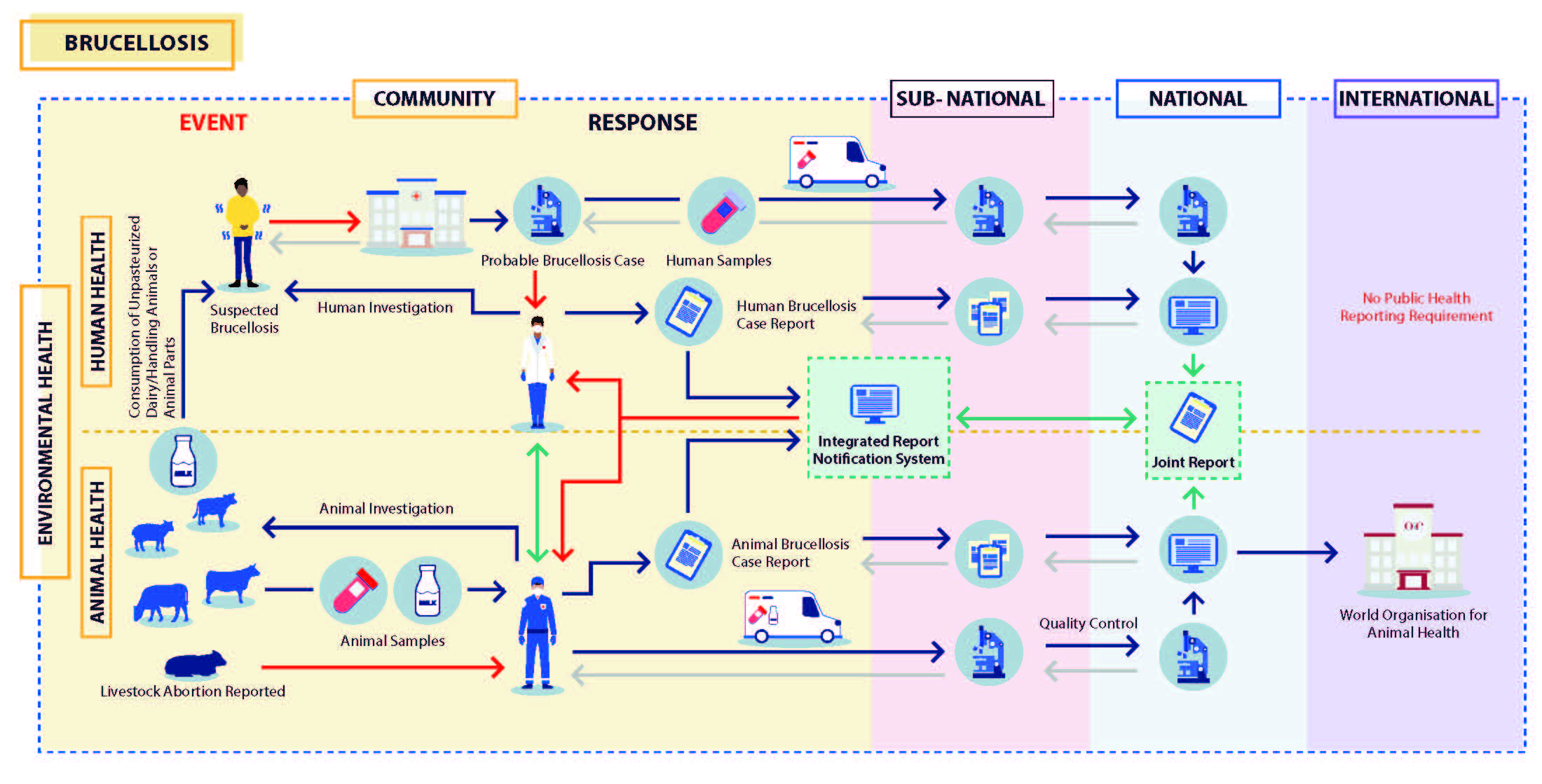


**Figure S2. Brucellosis.** Brucellosis is an economically significant bacterial zoonoses that is commonly endemic in livestock. In animals, infection can cause abortion storms, reproductive failure, premature births, and/or decreases in milk production. Human infections are usually acquired by consuming unpasteurized/raw dairy products, or by direct contact with infected animals or animal tissues. Human infection can result in febrile illness, fatigue, and muscle/joint pain. To facilitate response and control, an Integrated Report Notification System which intakes both human and animal case reports and notifies other relevant sectors of events is one possible mechanism that can facilitate community and sub-national level One Health linkages. At the national level, joint reporting can ensure that control efforts, which largely rest on the livestock sector, are endorsed and supported by data from all relevant sectors.


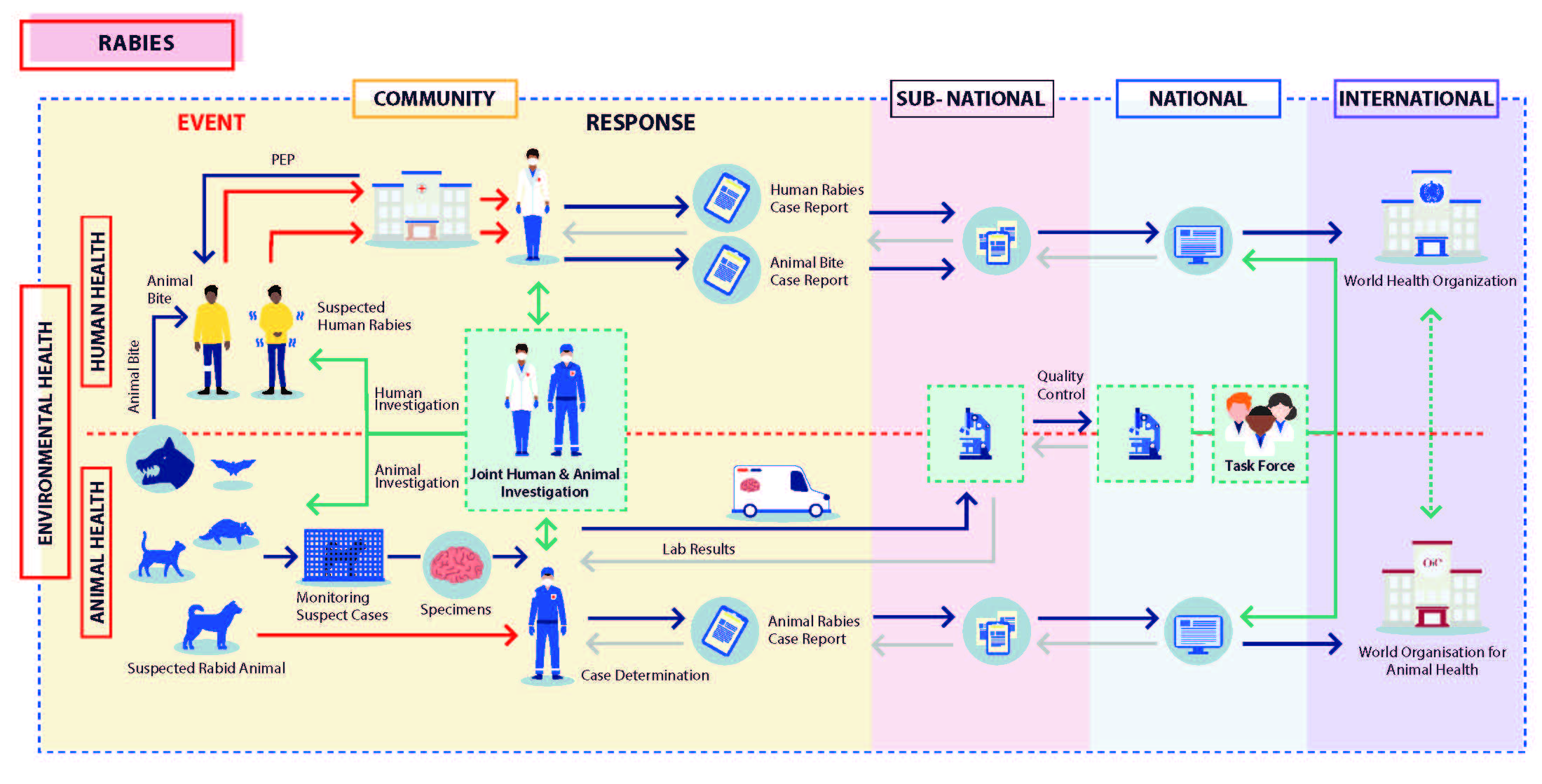


**Figure S3. Rabies.** Rabies is a highly fatal viral zoonoses that has no effective treatment after symptom onset. In many low-resource countries human rabies vaccine can be costly and difficult to obtain; to balance the lethality of the virus with cost and availability of vaccine, human and animal health sectors can institute a One Health-based system termed Integrated Bite Case Management (IBCM) to improve the efficiency of vaccine usage. IBCM systems are usually triggered by a bite event or clinically suspected rabid animal, which when recognized results in a field investigation to assess the animal and any people it may have exposed. The results of IBCM investigations result in tailored risk-assessments and medical advice for those exposed. Since post-mortem rabies diagnostic assays are equivalent for humans and animals, laboratory systems for rabies may also implement a One Health approach by accepting samples submitted from either sector. Finally, it is recommended that data is shared between sectors at the sub-national and national levels to ensure that rabies control and elimination programs channel resources according to the situation in both humans and animals.


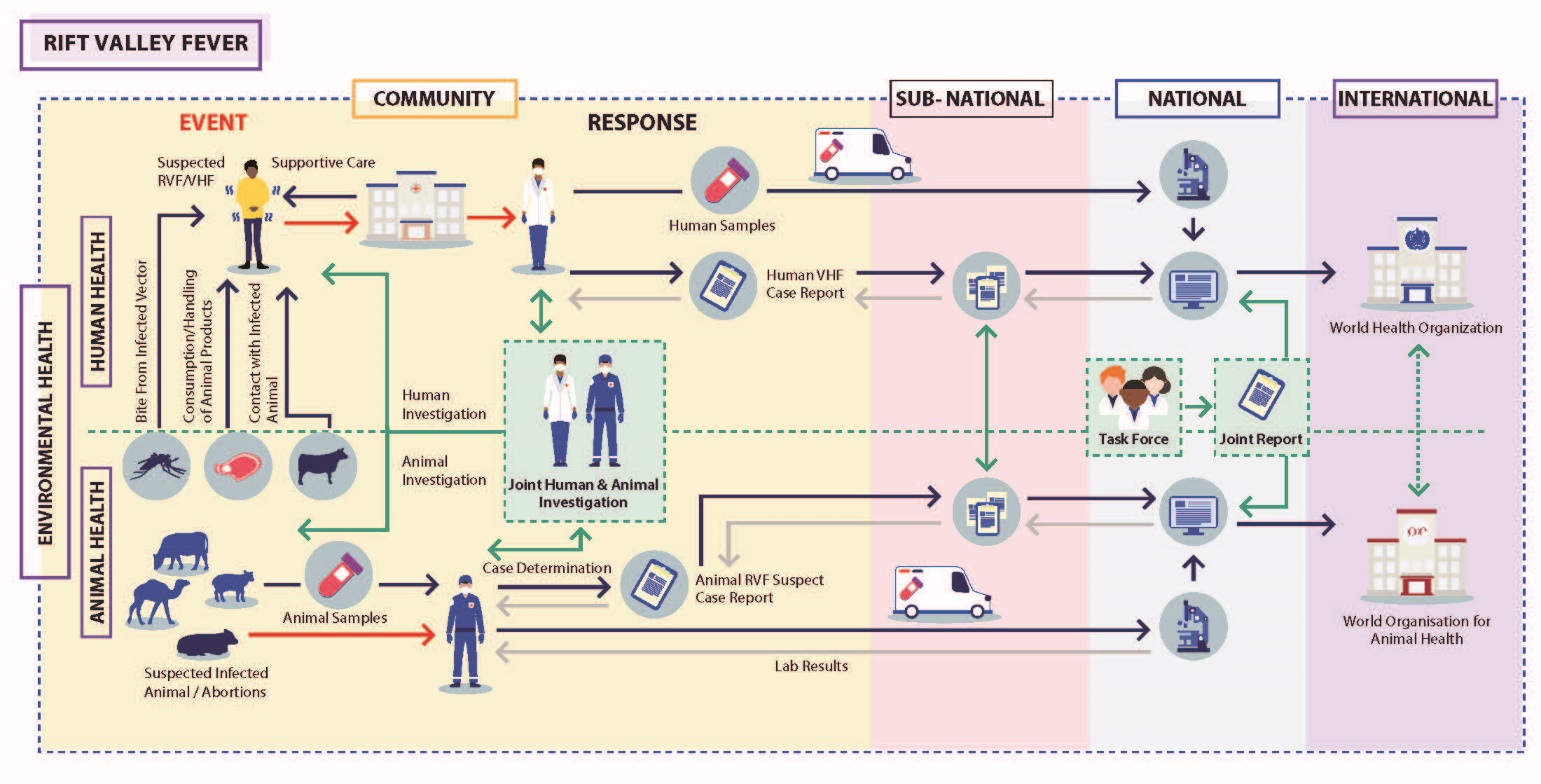


**Figure S4. Rift Valley Fever.** Rift Valley fever (RVF) is an acute, febrile viral disease most commonly observed in domesticated animals. Triggering events for identification of RVF in animals include illness and mass abortions in livestock. Animal outbreaks (“epizootics”) can lead to epidemics in human populations. Humans may have no symptoms or a mild illness associated with fever, but cases can be fatal. Symptoms can include ocular disease, encephalitis and hemorrhage in severe cases. Laboratory diagnosis is the same for both humans and animals and blood samples should be collected for rapid testing. Due to the highly transmissible nature of RVF, diagnostic testing is only appropriate in laboratories with adequate biosafety and training protocols. Effective response to both human and animal RVF cases requires an integrated One Health response to characterize the extent of the epizootic and implement control measures, health communication and intervention strategies. Where resources permit, a mosquito vector investigation component is also highly encouraged to gain a comprehensive understanding of the outbreak event.


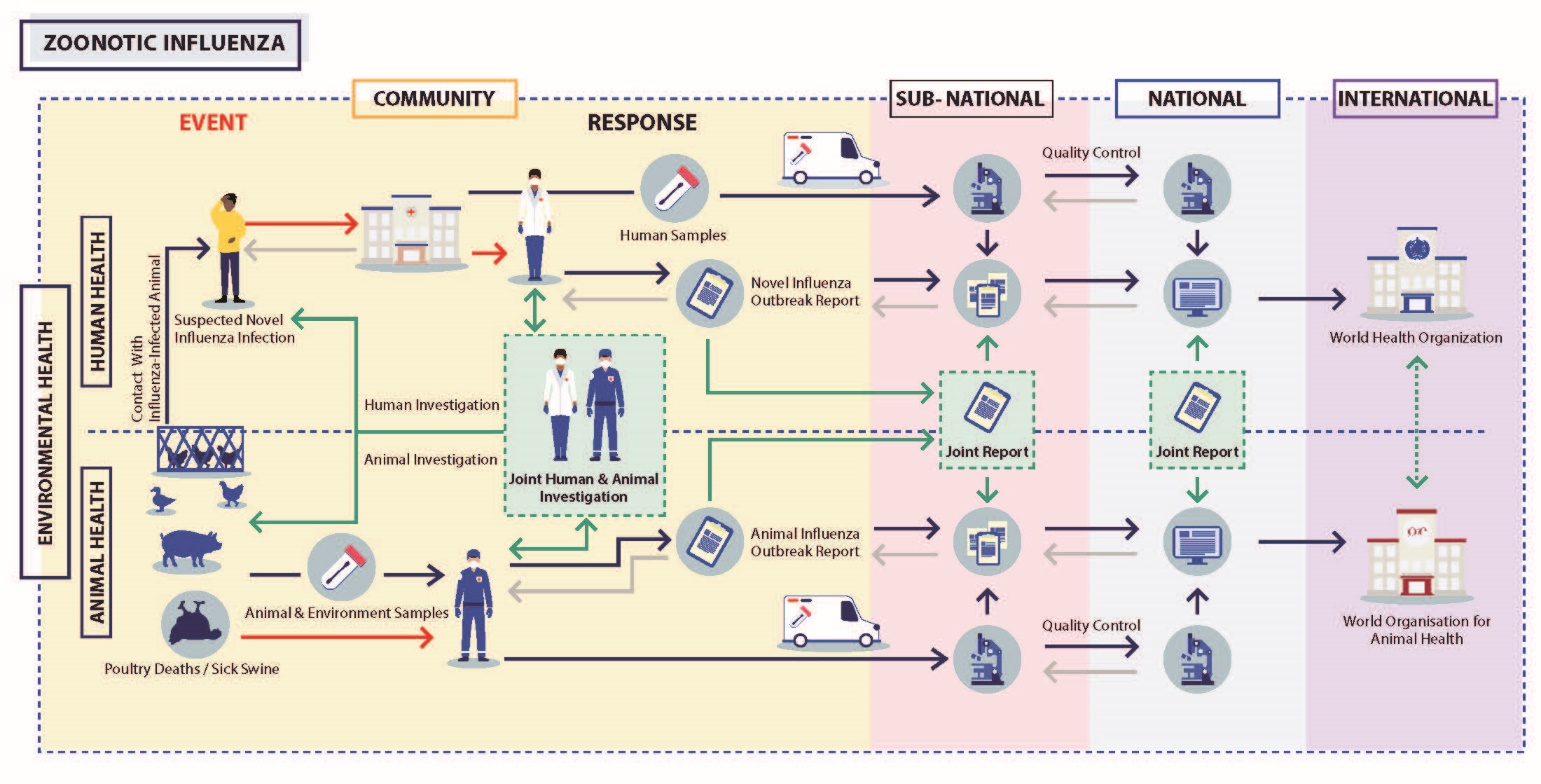


**Figure S5. Influenza.** Influenza (flu) is a contagious respiratory illness caused by influenza viruses. Influenza A viruses are found in humans and many different animals, including ducks, chickens, pigs, whales, horses, seals, dogs and cats. A few avian and swine influenza A viruses are zoonotic and capable of causing occasional human infections. Influenza pandemics happen when zoonotic influenza A viruses emerge that are able to infect people easily and spread from person to person in an efficient and sustained way. Effective surveillance that rapidly detects and responds to both animal disease outbreaks and sentinel human infections may prevent potential human pandemics. An effective One Health zoonotic influenza surveillance strategy should include joint investigations of human and animal infections along with joint reporting.
